# Design and Development for Capacitive Humidity Sensor Applications of Lead-Free Ca,Mg,Fe,Ti-Oxides-Based Electro-Ceramics with Improved Sensing Properties via Physisorption

**DOI:** 10.3390/s16071135

**Published:** 2016-07-21

**Authors:** Ashis Tripathy, Sumit Pramanik, Ayan Manna, Satyanarayan Bhuyan, Nabila Farhana Azrin Shah, Zamri Radzi, Noor Azuan Abu Osman

**Affiliations:** 1 Centre for Applied Biomechanics, Department of Biomedical Engineering, University of Malaya, Kuala Lumpur 50603, Malaysia; ayanbabu@gmail.com; 2 Department of Electronics & Instrumentation Engineering ITER, Siksha ‘O’ Anusandhan University, Bhubaneswar 751030, India; satyanarayanbhuyan@soauniversity.ac.in; 3 Department of Paediatric Dentistry & Orthodontics, Faculty of Dentistry, University of Malaya, Kuala Lumpur 50603, Malaysia; nabilafarhana.shah@gmail.com (N.F.A.S.); zamrir@um.edu.my (Z.R.)

**Keywords:** relative humidity, water absorption, porous, hydrophilicity, response, recovery, stability, mechanism

## Abstract

Despite the many attractive potential uses of ceramic materials as humidity sensors, some unavoidable drawbacks, including toxicity, poor biocompatibility, long response and recovery times, low sensitivity and high hysteresis have stymied the use of these materials in advanced applications. Therefore, in present investigation, we developed a capacitive humidity sensor using lead-free Ca,Mg,Fe,Ti-Oxide (CMFTO)-based electro-ceramics with perovskite structures synthesized by solid-state step-sintering. This technique helps maintain the submicron size porous morphology of the developed lead-free CMFTO electro-ceramics while providing enhanced water physisorption behaviour. In comparison with conventional capacitive humidity sensors, the presented CMFTO-based humidity sensor shows a high sensitivity of up to 3000% compared to other materials, even at lower signal frequency. The best also shows a rapid response (14.5 s) and recovery (34.27 s), and very low hysteresis (3.2%) in a 33%–95% relative humidity range which are much lower values than those of existing conventional sensors. Therefore, CMFTO nano-electro-ceramics appear to be very promising materials for fabricating high-performance capacitive humidity sensors.

## 1. Introduction

Humidity is a physical parameter which describes the degree of dryness of the atmosphere. It is an extremely important factor in many sectors, including hospitals, textile industries, laboratories, storage rooms for computers, food processing industries, art museums, shopping malls, libraries, exhibition centres, and so on. It is thus necessary to develop highly sensitive humidity sensor materials having good reliability, good linearity, long-term stability, rapid response and recovery, and small hysteresis [1]. Recent studies have attempted to develop more sophisticated humidity sensors by manipulating several sensor characteristics such as refractive index, frequency range, capacitance, impedance, and sensing mechanisms [2,3,4]. These characteristics are strictly determined by the sensing medium material, porosity, surface area, and pore size distribution. To date humidity sensors have been produced using various materials, including electrolytes [5], organic polymers [6] and ceramic materials [7]. The most unique properties of ceramic materials are their relatively high thermal, chemical and mechanical stability [8,9], which make them very suitable potential candidates in sensor applications, and the excellent water adsorbing and desorbing properties of metal oxide ceramics such as TiO_2_ [10], ZnO [11], ferrite [7], Al_2_O_3_ [12,13] and (Ba,Sr)TiO_3_ [14,15] have been extensively utilized in humidity sensors. In particular, some porous ceramic-based capacitive sensors can remain stable under high humidity conditions, even at elevated temperature, while showing good sensitivity [16,17]. Some precision capacitive methods using ceramic materials also have shown rapid dynamic responses and high temperature compensation [18,19]. They can also have nonlinear hysteresis characteristics and require only temporary heating. However, due to the property of water molecule chemisorption over a wide humidity range, many ceramic-based humidity sensors still cannot achieve sufficient sensitivity, reversibility or resistance stability. This limitation hinders their use in the development of advanced applications.

Perovskite-structured (ABO_3_) oxide materials [20,21] have widely been used as semiconductors, high temperature ionic conductors, ferroelectrics, dielectrics, as well as humidity sensors, for different applications. In perovskite-structured materials, the A-site atoms are susceptible to humidity [22], and by partially substituting the A-site atoms with other elements, e.g., rare earth cations, their humidity sensitivity can be enhanced [23]. The mixed ionic and electronic conducting properties of perovskite-structured oxides provide advantages of higher selectivity, activity and stability than seen with simple transition metal oxides. Their potential applications as cathode materials in oxygen permeation membranes (OPMs) for air separation [24], catalysts for hydrocarbon oxidation reactions [25,26], solid oxide fuel cells (SOFCs) [27] and catalytic membrane reactors (CMRs) for syngas production [28] have also popularized their use among researchers.

Most of these materials are lead (Pb)-based compounds such as lead magnesium niobate (PbMg_1/3_Nb_2/3_O_3_), lead titanate (PbTiO_3_) and lead zirconate titanate (PbZr_1−x_Ti_x_O_3_) [29,30,31] and so on. Pb-based ceramics such as PbTiO_3_, Ca_x_Pb_1−x_TiO_3_, Li-Ca_0.3_Pb_0.65_TiO_3_ have been used as humidity sensors in many studies [32,33], but despite their substantial advantageous dielectric, capacitive and ferroelectric properties, these Pb-based compounds are toxic and hazardous to environment and human health. Furthermore, the manufacturing and machining wastes of the electronic components of various devices cause lots of pollution; in particular Pb-based materials cannot be easily recycled [34,35]. Emphasis has therefore been placed on finding eco-friendly Pb-free perskovite materials with high humidity sensitive electrical properties comparable to those of Pb-based ones, such as barium titanate (BaTiO_3_)-based materials or calcium titanate (CaTiO_3_, CTO). CTO is now a common humidity sensing ceramic widely used in the form of porous sintered bodies [31]. The humidity-dependent electrical properties of CTO can be altered by substituting various either isovalent or heterovalent cations separately or simultaneously at the Ca or Ti sites. In this context, the effect of isovalent substitutions on the electrical properties and transition temperatures of CTO have been investigated [36,37]. To improve the electrical characteristics valence-compensated solid solutions, i.e., simultaneous heterovalent substitutions with equimolar concentrations of other species at the Ca and Ti sites of compounds such as Ca_1−x_La_x_Ti_1−x_Cr_x_O_3_, and Ca_1−x_Bi_x_Ti_1−x_Cr_x_O_3_ have also been studied [38,39,40]. However, to our knowledge the performance of heterovalent substitutions with non-equimolar concentration at the Ca and Ti sites of CTO in different humidity environments has not been investigated properly.

The present article therefore aimed to synthesize Mg^2+^, Fe^2+^ doped CTO electro-ceramic (Ca,Mg,Fe,Ti-Oxide) (CMFTO)-based humidity sensor nanomaterials with rapid responses and recovery times. The CMFTO electro-ceramics were prepared by an innovative solid-state step-sintering process to get the desired morphology with lower density and high porosity. This facile synthesis process is convenient, eco-friendly and easily controlled, compared to other synthesis techniques [29,30,31,32,33]. In this route, cheap raw materials like solid titania (TiO_2_) and other metal ions from magnesium carbonate (MgCO_3_), hematite (Fe_2_O_3_), and calcium oxide (CaO) have been taken in solution form. This offers an advantage over other expensive synthesis techniques where the prominent sources of TiO_2_ are titanium alkoxide, oxynitrate, or chloride. This route is also very useful to obtain a homogeneous and fine precursor powder. In this unique synthesis technique, a rapid heating rate, proper isothermal holding method, and considerably reduced processing time and temperature are the main advantageous features compared to conventional ceramic processing methods. This technique can also be implemented in industrial production to reduce production costs and eliminate hazardous by-products. Additionally, TiO_2_, MgCO_3_, Fe_2_O_3_, and CaO nanopowders are generally nontoxic, and possibly biocompatible, and thus, they have been used in many different biomedical applications. We therefore aimed to design and develop an improved sensitive humidity sensor using a high pressure treated solid-state step-sintering technique and starting from inexpensive Pb-free nanomaterials.

## 2. Experimental Section

### 2.1. Preparation of the Sensing Nanomaterial

A perovskite-structured polycrystalline sample of CMFTO was prepared by our solid-state step-sintering technique using high-purity ingredients such as MgCO_3,_ Fe_2_O_3,_ TiO_2_ and CaO (99.9%, Fisher Scientific Ltd., Selangor, Malaysia) in a desired stoichiometry. The dry mixed oxide powders of all the ingredients (CaO, MgCO_3_, Fe_2_O_3_ and TiO_2_ of 11.59, 17.4, 21.9, and 49.11 wt%, respectively) were milled with 70% alcohol in a ball-mill (PM200, Retsch, Düsseldorf, Germany) with a sample to ball ratio of 1:200 (*w*/*w*) for 72 h. After ball-milling, the milled CMTFO slurry was dried at 105 °C in a convection oven (OF-11E, Lab Companion, Seoul, Korea) for 6 h to remove the excess moisture, water and alcohol. The dry-powder was cold pressed into pellets of 10 mm diameter and 1.2 mm of thickness under uniaxial high pressure (450 MPa) at 25 °C into a cylindrical mould using a hydraulic press (GS15011, Graseby Specac, Kent, UK). Polyvinyl alcohol (PVA, <2 wt%) was used as binder to reduce the brittleness of the pellets during pelletization. The binder was burnt out at high temperature during sintering. It also helped the sintered pellets to achieve porous morphology [41]. The step-sintering technique was used to sinter the ceramic pellets at different temperatures such as 450, 650, 850, and 1050 °C in a programmable furnace (XY1600, Nanyang Xinyu Furnaces, He’nan, China) under a normal atmosphere to control the particle size, pore size, as well as porosity up to a desired range. The complete steps of different sintering temperature are illustrated in Table 1.

**Figure 1:**
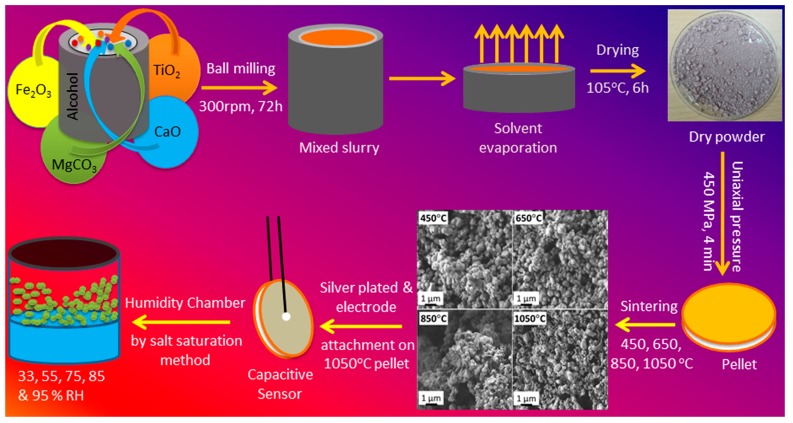
Flow chart for sensor fabrication with the morphology at different sintering temperature.

A schematic flow chart of the synthesis processes is depicted in Figure 1. A probable complete chemical reaction after sintering at 1050 °C is depicted in the following reaction [41]:

CaO + MgCO_3_ + 4Fe_2_O_3_ + 4TiO_2_ + 0.5O_2_ ↑ → Fe_2_MgTi_3_O_10_ + CaTiO_3_ + 2Fe_3_O_4_ + O_2_ ↑ + CO_2_ ↑

### 2.2. Fabrication of Humidity Sensor

The uniaxially compressed sintered and unsintered pellets were polished with emery paper and cleaned with absolute alcohol, followed by drying at 105 °C before using them as sensors. Thereafter, silver (Ag) electrodes were screen printed on both sides of the each pellet followed by drying at 80 °C for 30 min. Then, copper (Cu) wire was connected to the Ag-electrodes as conducting wire. Finally, the sensor was aged at 150 °C in air for 60 min.

### 2.3. Physical Characterizations

The microstructural analysis was carried out by field emission scanning electron microscopy (FESEM, AURIGA, Carl Zeiss, Jena, Germany). Pore size distribution (PSD) and relative cumulative frequency (RCF) of the pores were evaluated from the corresponding FESEM images and analyzed with the ImageJ software. An X-ray diffractometer (Empyrean, PANalytical, Almelo, The Netherlands) was used to obtain the different X-ray diffraction (XRD) patterns of the developed electro-ceramics using Cu-Kα radiation in the 2θ range of 20°–50°. Density (*ρ*, g/cc), open porosity (%), and water absoption (%) ability of electro-ceramics were measured following Equations (1)–(3), respectively, with the help of a modified Archimedes’ principle as highlighted in our previous studies [42,43]:

(1) $$\rho =\frac{W_{bi}}{W_{ai}-W_{di}}\times \rho_{water}^{25\;{}^{\circ}C}$$

(2) $$P_{open}=\frac{W_{ai}-W_{bi}}{W_{ai}-W_{di}}\times 100$$

(3) $$\text{Absorbed water}=\;\frac{W_{ai}-W_{bi}}{W_{bi}}\times 100\;$$

where *W*_bi_, *W*_di_ and *W*_ai_ represent the initial dry weights of the samples in air before immersion in water, in water during immersion in water, and in air after immersion in water, respectively. The resolution of the weighing machine was ±0.0005 g. At least five identical specimens were prepared to calculate the standard deviation (SD) of all the sintered samples, where $\rho_{\text{water}}^{25\;{}^{\circ}C}$ represents the water density at tested temperature 25 °C. Water contact angle (WCA) of the pellet samples was measured using sessile contact angle meter (OCA15E, Data Physics Instruments GmbH, Filderstadt, Germany) at room temperature.

### 2.4. Humidity Sensor Measurements

**Figure 2:**
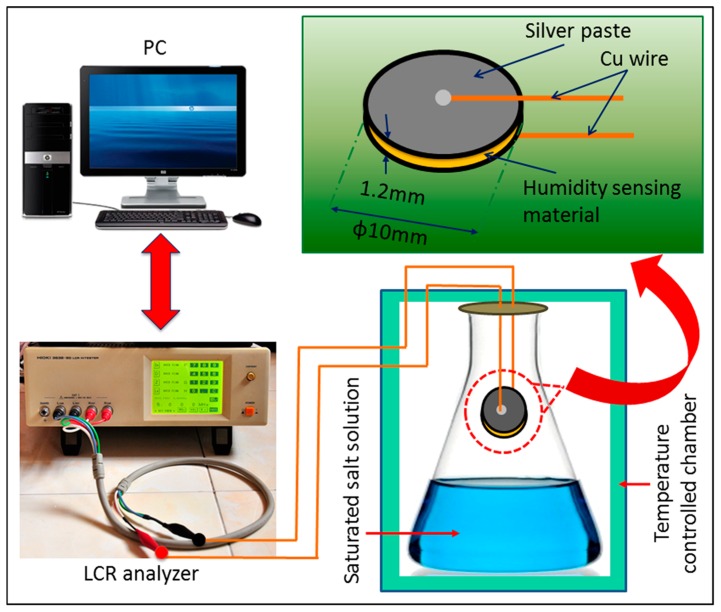
Experimental setup for the measurement of the capacitive humidity response of the electro-ceramic based sensors.

The RH-dependent capacitive response of the CMFTO electro-ceramic was determined at 25 °C while the RH was changed from 33% to 95%. First, the final aged sensors were stabilized at 1 V voltage in 95% RH for 24 h. Ambiences with different humidity levels were then created by silting a series of standard saturated salt solutions (MgCl_2_, Mg(NO_3_)_2_, NaCl, KCl and KNO_3_) in conical flasks with stoppers, to produce environments with relative humidity (RH) values of 33%, 55%, 75%, 85% and 95% at 25 °C, respectively as described in our previous report [41]. The capacitance response of the CMFTO electro-ceramic with RH was measured by impedance spectroscopy (IS) (3532-50 LCR Hi tester, Hioki, Ueda, Japan) at 25 °C in a temperature controlled chamber (Memmet, Naluri Scientific, Schwabach, Germany) with a resolution of ±5 °C over a frequency range of 10^2^ Hz–10^6^ Hz. All the humidity sensing measurements were carried out under normal atmospheric pressure. The measurement setup is depicted in Figure 2.

## 3. Results and Discussion

### 3.1. Structural and Morphological Characterization

**Figure 3:**
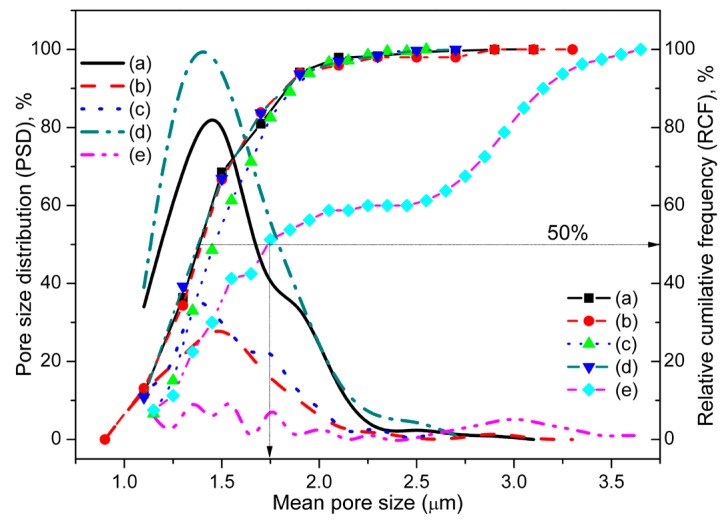
Pore size distribution (PSD) and relative cumulative frequency (RCF) of (**a**) unsintered and sintered at (**b**) 450 °C, (**c**) 650 °C, (**d**) 850 °C and (**e**) 1050 °C materials measuring from the electron micrographs using ImageJ.

Pore size and pore size distribution (PSD) are two of the most important parameters for all humidity sensor nanomaterials. The PSD and the relative cumilative frequency (RCF) of all the CMFTO ceramics are illustrated in Figure 3. The PSD of the unsintered and sintered nanomaterials was calculated from the corresponding FESEM images (see Figure 1 and also Figure S1 in the Supplementary Information) using the ImageJ software. Average particle sizes of all the nanomaterials are given in the Supplementary Information. Most of the pores remained at less than 1.5 μm at sintering temperatures up to 650 °C. Interestingly, at 850 °C and 1050 °C, bimodal and trimodal pores were clearly revealed in PSD plots, suggesting that higher sized open pores are formed at higher sintering temperatures. However, a few large size pores were also found up to 450 °C owing to the collapse of loose bonds on the surface of the pellets. The 50% RCF of the sample sintered at 1050 °C is projected to have a pore size of 1.75 μm, which is significantly higher compared to unsintered and other sintered (<1.4 μm) materials (see the thin projected arrows in Figure 3). This RCF result is clear evidence for selecting the sintering condition at 1050 °C as the nanomaterial with most potential for our futher studies. Furthermore, the main three pore size modes (3 μm, 1.75 μm and less than 1.5 μm) seen in the material under the 1050 °C sintering condition were obtained due to three different types of cluster developed by three different structuresd phases—armalcolite, perovskite and ferrite—which was confirmed by our XRD study (see Figure S2 in the Supplementary Information). A wide pore size distribution can be used to realize a humidity sensor capable of operating over a wider range of humidity. The pore size distribution has also been widely considered as an important function for better sensitivity in a particular humidity range. The advantage of porosity is that at a particular temperature and relative humidity (RH), water condensation occurs in pores [44]. In addition, the lower pore size (nearly 100 nm) of TiO_2_-based humidity sensor materials formed nanogaps and hence showed worse sensing properties such as long recovery times compared to the larger pore size of 4.5 μm [45]. The presence of crystalline peaks of anatase phase TiO_2_ (PDF: 98-015-4609) along with Fe_2_O_3_ (PDF: 01-084-0308), MgCO_3_ (PDF: 00-002-0905) and CaCO_3_ (PDF: 01-072-1650), which was converted from CaO during the wet-ball-milling, in the unsintered nanomaterial has already been reported in our previous study [41]. XRD patterns of all the ceramic samples are depicted in Figure S2. A new armalcolite phase (Fe_2_MgTi_3_O_10_, as PDF: 00-013-0353) was formed after sintering at 450 °C. Then, another phase of perovskite CaTiO_3_ (PDF: 00-008-0092) with sharp small (400) at 2*θ* = 23.43° and strong (440) at 2*θ* = 33.29° crystalline peaks was developed after increasing the temperature above 650 °C. The more intense crystalline peaks (230) at 2*θ* = 32.71° of Fe_2_MgTi_3_O_10_ and (440) of CaTiO_3_ become stronger after sintering at 1050 °C. A significant shift of a (110) peak of Fe_2_O_3_ (2*θ* = 35.83°) strongly indicates the formation of magnetic Fe_3_O_4_ (2*θ* = 35.70°), which may further stimulate the use of this material in remote sensing and humidity sensor applications [46].

On the other hand, FESEM images of the materials depicted in Figure S1a–d reveal a uniform submicroporous structure for the ceramic nanomaterials. It has been found that the structure of CMFTO is affected by the sintering temperature. An increase in sintering temperature produced significant changes in the microstructure (Figure S1). The average particle size of the unsintered ceramic was found to increase at the sintering temperatures of 450 °C and 650 °C. However, the growth rate was impeded by changing sintering steps at 850 and 1050 °C without much change in the total pore distribution. A smaller size (typically < 100 nm) of new phase of CaTiO_3_ particles was noticed in Figure S1d. With an increase in sintering temperature, it was found that the grain size was slightly increased and some grains were colligated together with each other (Figure S1). This interconnected grain texture could have resulted from melting and by a controlled crystal growth mechanism. This would help the present CMFTO material create porous microstructure and thus, form materials to adsorb or desorb water molecules under humid conditions.

**Figure 4:**
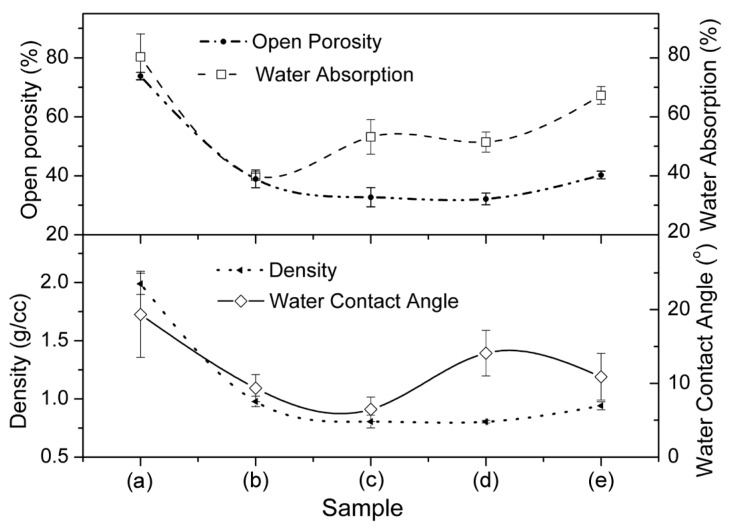
Density, open-porosity, water absorption and water contact angle (WCA) of (**a**) unsintered and sintered at (**b**) 450 °C; (**c**) 650 °C; (**d**) 850 °C and (**e**) 1050 °C ceramic samples.

The bulk density, open porosity, water absoption and WCA of all the pellet samples are depicted in Figure 4. The WCAs of all the sintered pellets are significantly lower than those of unsintered ones. The open porosity of all these materials, calculated using their bulk density, was found to be lower than their closed porosity. The open porosity of unsintered and sintered at 1050 °C materials were measured to be 73.84% ± 1.24% and 40.26% ± 1.33%, respectively. On the other hand, the density of unsintered material was substantially reduced (by almost half) from 1.989 ± 0.091 g/cc to 0.941 ± 0.035 g/cc after sintering at 1050 °C. This is possible due to a lattice diffusion phenomenon during sintering while maintaining substantial porosity [47]. Lower density values of these pellets compared to their commercial ingredients indicates the presence of a higher amount of porosity. This study also confirms that a new phase, CaTiO_3_ (see Figure S1) was formed by a lattice diffusion mechanism [41]. The total porosity of the materials was evaluated from their water absorption (see Figure 4). The higher amount of water absorption (nearly 67%) in the material sintered at 1050 °C confirms the presence of uniform porosity that was revealed in the FESEM image (see Figure S1e). This material also showed the lowest density and a larger porosity (compared to the sintering condition at 805 °C) with a wide pore size distribution. A larger pore size (see Figure 3) and high open porosity (see Figure 4) imply a much more active surface towards water vapor and thus, the sensitivity of the capacitance to humidity will be increased. Large pores are necessary for a rapid response to exhibit easy adsorption and desorption or condensation of water vapour [48]. Since the sample sintered at 1050 °C showed a higher amount of water absorption (~67%) and higher porosity with lower density compared to the material unsintered or sintered at or below 850 °C, the material sintered at 1050 °C would be considered a most favourable candidate for humidity sensor analysis in our further studies [49,50,51,52].

### 3.2. Humidity Sensing Measurements

**Figure 5:**
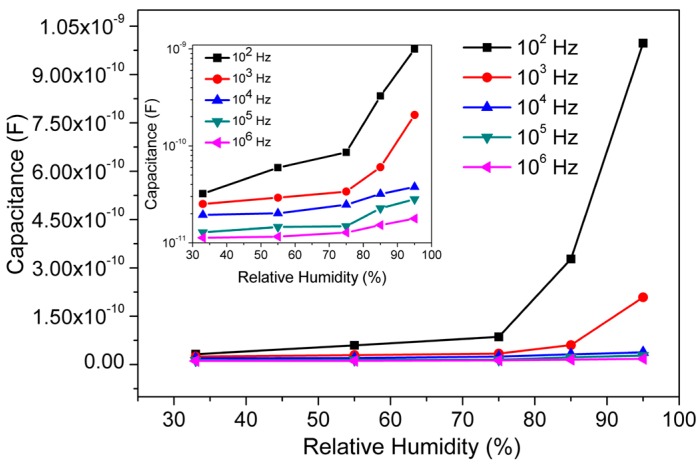
The response curves of the capacitance versus relative humidity (RH) at different frequencies of CMFTO electro-ceramic at 25 °C. Inset image represents the variation of capacitance with RH at 25 °C at different frequency in logarithmic scale (log(*C*) vs. % RH). Note: the capacitance increases monotonically with % RH at different frequencies, but increased rate is faster at 10^2^ Hz.

All the aforementioned physical properties favour considering the porous CMFTO electro-ceramic sintered at 1050 °C to be the best hydrophilic material among the all ceramics developed in the present study. The hydrophilic characteristics of its surface with meso- and submicropores should be helpful for improving the water molecule adsorption/desorption process and hence, enhance the sensitivity of humidity sensors. To investigate the effect of frequency on sensing characteristics of the CMFTO electro-ceramic based sensor, its capacitance values have been calculated under different humidity environments with various test frequencies such as 10^2^, 10^3^, 10^4^, 10^5^ and 10^6^ Hz at 25 °C and the results are depicted in Figure 5. It has been observed that higher frequencies lower the capacitance; for example, at 95% RH the highest capacitance value of 9.974 × 10^−10^ F at 10^2^ Hz and lowest C of 1.780 × 10^−11^ F at 10^6^ Hz have been observed. It has also been found that when the RH increases from 33% to 95%, the capacitance value increases monotonically. The plots of the inset image of Figure 5 are a better representation of the change in C-values as a function of % RH (33%–95%) at different frequencies (10^2^–10^6^ Hz). This suggests that the capacitance is an effective physical parameter in order to evaluate the sensor response.

**Figure 6:**
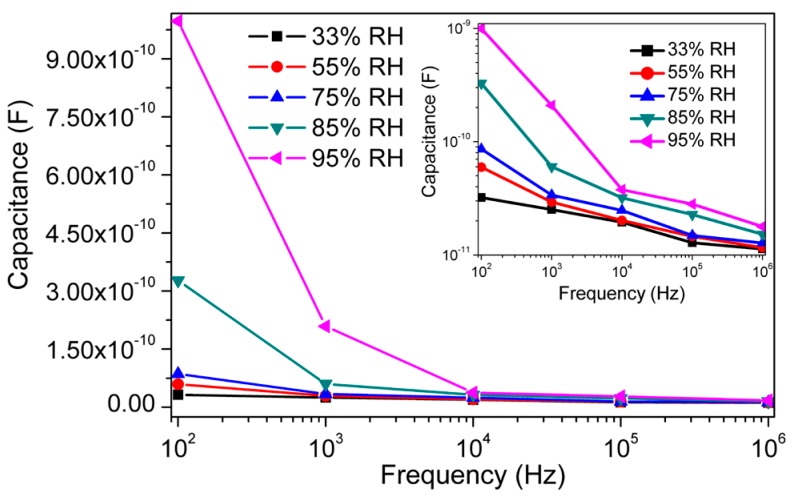
The variations of capacitance with frequency at different humidity condition (33%–95% RH) for CMFTO based humidity sensor at 25 °C. Inset image represents the variation of capacitance with frequency at different RH in logarithmic scale (log(*C*) vs. log(RH)). Note: The value of capacitance increases with increased % RH, but decreases with increased frequency. The decreased rate is faster in lower frequency (<10^4^ Hz) and higher humidity range (>85% RH).

The relations between capacitance and frequency at 25 °C of the present capacitive-type sensor at different RH values are depicted in Figure 6. It has been observed that the capacitance (C)-value decreases with increasing frequency and the rate of decrease is faster at higher RH. The capacitance value increases significantly with % RH in the lower frequency range (below 10^4^ Hz) but in a higher frequency range, the capacitance value is small and hardly changes with humidity. Generally in the ideal capacitive sensor, the C-value is independent of applied frequency. However, in low humidity environments, the sensing material absorbs a small amount of water, which could be considered as an ideal situation. Due to the absorption of water molecules, the sensing material possesses a leak conduction (γ) [53]. The capacitance (C) of the material with leak conduction can be expressed by a relation as shown in Equation (4) [53,54]:

(4) $$C={є}^{*}C_{0}=\left({є}_{r}-i\frac{\gamma }{\omega {є}_{0}}\right)C_{0}$$

where є*, *C*_0_ and *є*_r_ are the complex dielectric constant, capacitance and relative dielectric constant of an ideal capacitor, respectively; *ω* is the angular frequency, *γ* is the conductance and *є*_0_ is the permittivity at free space. From Equation (4), it can be noticed that the capacitance of the sensing material is inversely proportional to frequency *ω* and directly proportional to *γ*. Hence, the *C*-value decreases with increasing frequency and this relation becomes more prominent under higher RH conditions. In addition, *γ* is attributed to the physisorption of water molecules on the surface of porous CMFTO electro-ceramic. Here, *γ* increases with increasing of % RH and as a result, *C*-value increases with rising RH with respect to *ω*.

**Figure 7:**
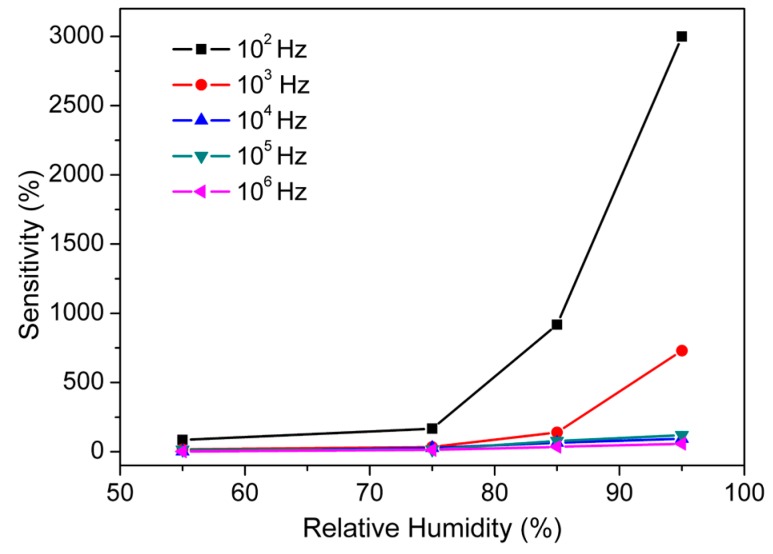
The sensitivity (%S) response of CMFTO based capacitive sensor with % RH at different test frequencies at 25 °C. Note: the sensitivity increases monotonically with % RH at different frequencies, but the value of sensitivity is highest (~3000%) at 10^2^ Hz. Hence, 10^2^ Hz is considered as the most suitable frequency for the further analysis.

To explain the humidity-dependent capacitive characteristics of CMFTO electro-ceramics, the device sensitivity (*S*) has been calculated by using Equation (5) [55]:

(5) $$S=\frac{C_{RH}-C_{33}}{C_{33}}\times 100\%$$

where *C*_33_ and *C*_RH_ stands for the capacitances measured at 33% RH and at a certain RH level, respectively. Figure 7 shows that the applied test frequency has a high influence on the sensitivity of humidity sensors. It has been also observed that the values of *C* (Figure 5) and *S* (Figure 7) with % RH (in the range 33%–95%) increase with decreasing test frequencies (from 10^6^ to 10^2^ Hz). The *C*-value increased from 3.2183 × 10^−11^ F to 9.9741 × 10^−10^ F with a “*S*” of ~3000% at the signal frequency of 10^2^ Hz but when the test frequency is 10^5^ Hz, the capacitance varied from 1.2832 × 10^−11^ to 2.8174 × 10^−11^ F with “*S*” of ~120% across the RH range of 33%–95%, respectively. The sensitivity of our developed sensor is higher than that of some other ceramic based humidity sensors reported elsewhere (for example, ~2900% for porous ZnAl_2_O_4_ spinel nanorod, 85% for silica nanoparticles aerogel thin films, ~966% for SiC nanowires, ~800% for thermally carbonized porous silicon, ~500% for alumina nanowire, 1490% for (PEPC + NiPc + Cu_2_O) and so on based humidity sensors) [16,56,57,58,59,60]. Therefore, in our further study the 10^2^ Hz would be considered as best test frequency for the analysis of sensor characteristics.

**Figure 8:**
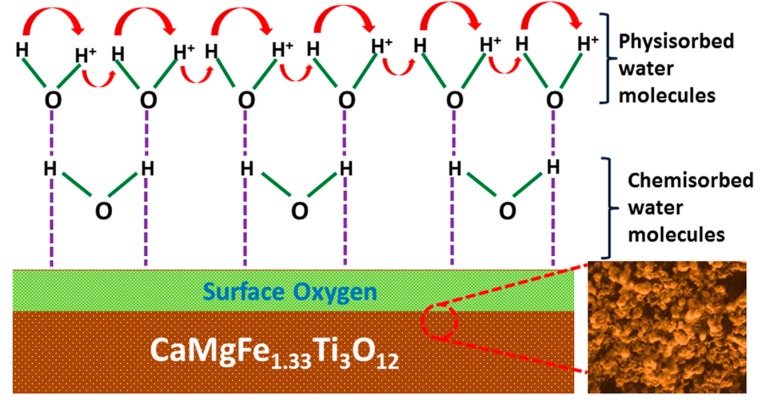
Schematic representation of the humidity sensing mechanism of CMFTO electro-ceramic at different humidity environment. Note: the adsorption of water molecules on CMFTO nanoceramic is characterized by two processes. The first-layer water molecules (at lower humidity) are attached on the CMFTO electro-ceramic through two hydrogen bonds. As a result, the water molecules are not able to move freely and thus, the impedance value increases. In contrast, from the second layer (at higher humidity), water molecules are adsorbed only through one hydrogen bond. Hence, the water molecules are able to move freely and thus, the impedance value decreases. This insists to increase the capacitance value.

The humidity sensing mechanism of the CMFTO electro-ceramic could be explained by adsorption phenomena of water molecules and their effect on the capacitance variation of the system. The mechanism for humidity-dependent electrical characteristics of the oxide-based nanomaterial is not so clear to date. Therefore, a possible physical adsorption mechanism of water molecules for the humidity dependent electrical characteristics of the oxide based electro-ceramic nanomaterials is explained here. The relationship between the capacitance and the RH, can be interpreted by using Equation (4). On the other hand, the ionic conductivity increases with increasing RH [61], and as a result the capacitance value increases with RH as a function of frequency. In general, under low RH conditions, the water molecules are primarily physisorbed or chemisorbed onto the available active sites of the oxide based CMFTO electro-ceramic surfaces through double hydrogen bonding as indicated by the dotted-line in Figure 8. Due to the double hydrogen bonding, the water molecules are not able to move freely. Thus, more energy is required for the hopping transfer of protons between adjacent hydroxyl groups. Hence, the CMFTO electro-ceramic exhibits very high electrical impedance in the double hydrogen bonding regime. The protons in CMFTO ceramic which are hindered by discontinuous mobile layers, may generate leak conduction and thus increase the capacitance [61]. On the other hand, at higher % RH, second or multi physisorbed layers are formed by physisorption of water molecules onto the available active sites of the surface oxygen of the oxide-based CMFTO electro-ceramic through single hydrogen bonding. Owing to the single bonds, the water molecules become mobile and progressively more identical to those in the bulk liquid. With further increase of RH, the multilayer physical adsorption increases and as a result the physisorbed water molecules are ionized and produce a large number of hydronium ions (H_3_O^+^) as charge carriers due to the application of external electric field. In very high humidity conditions, the amount of water content increases and the physisorbed water layers gradually behave like normal condensed liquid. In this condition, the protons require very low energy for hopping between adjacent water molecules; as a result the ionic conductivity increases. This charge transport mechanism can be explained by a Grotthuss chain reaction (H_2_O + H_3_O^+^ → H_3_O^+^ + H_2_O) conductivity [62]. In addition to the above discussion, at higher RH, the physisorbed water penetrates into the interlayer of CMFTO electro-ceramic. As a result, the hydrolysis process becomes more effective with the functional groups of oxide based CMFTO electro-ceramic. Thus, more ions are generated due to the vigorous hydrolysis reaction, and these ions participate in the ionic conduction, and as a result the impedance value decreases [63]. In addition to the impedance, due to increase of water molecules, the capacitance value increases, which significantly alters the dielectric constants of the material. For instance, the dielectric constant of CMFTO electro-ceramic nanomaterial is calculated to be 233 at low RH (33% RH) and 5617 at high RH (95% RH).

**Figure 9:**
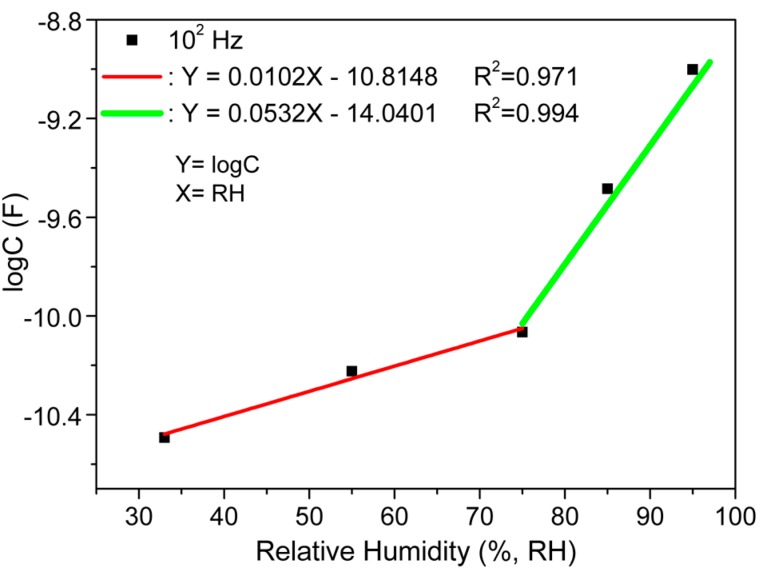
The transformed response curves of logarithmic capacitance (logC) vs. RH of CMFTO electro-ceramic based capacitive sensor. Note: first linear transformation curve (red-line) is well fitted by logC = 0.0102RH − 10.8148 in the RH range from 33% to 75% and the second linear transformation curve (green-line) is well fitted by the formula logC = 0.0532RH − 14.0401 at the higher humidity range (>75% RH). Here, regression, R^2^ represents a best fit of the curves to improve linearity.

The nonlinear characteristics on RH dependent capacitive response (Figure 5) might be a systematic error in standardization for practical applications of the present humidity sensor. Therefore, to overcome this nonlinearity drawback, an exponential function can be introduced to make the nonlinear response more linear [55]. Therefore, in the present study, a transformed logarithmic capacitive-RH response curve was generated and is depicted in Figure 9. It has been observed that two different slope-linear relations revealed in the logC vs. RH response curve intersect. This intersection point at 75% RH is considered as a critical point. It has been analyzed that the data could be well fitted linearly with a slope of 0.0102 and intersect at 10.8148 in negative logC-axis in the RH range from 33% to 75%, but in the higher humidity range (75%–95% RH), the slope of the linear fitted curve was found to increase to 0.0532 (see Figure 9). Here, the regression (R^2^) values of both the fitted curves were very close to 1 (more than 0.97), representing the best fit of the curves to improve linearity. The two different slopes of logC vs. RH response plot might be contributed to the change of the adsorption mode of water molecules from a monolayer chemisorption and the multilayer physisorption at low and high RH, respectively [9,64,65]. This linearity and sensitivity consideration of the logarithmic response curve represent a breakthrough for the CMFTO electro-ceramic as a suitable humidity sensing material.

The maximum difference in *C*-value between the humidification and desiccation curve is known as hysteresis. High hysteresis values have long been a major drawback in practical humidity sensor applications. The maximum hysteresis rate (*E*_max_) of our developed sensor has been calculated by using Equation (6) [55]:

(6) $$E_{max}=\frac{\Delta m}{Y_{FS}}\times 100\%$$

where, Δ*m* stands for the maximum hysteresis and *Y*_FS_ is the full scale output.

**Figure 10:**
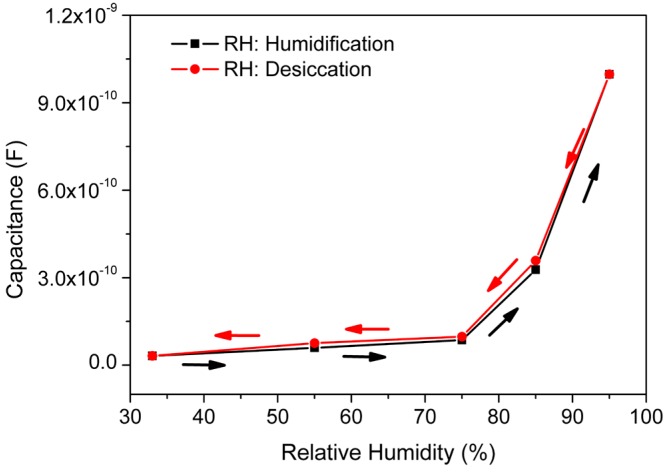
The hysteresis property of CMFTO electro-ceramic-based capacitive humidity sensor at 10^2^ Hz under 25 °C. Note: the value of hysteresis is extremely low (~3.2%) compared to other conventional capacitive sensors. The low hysteresis value is mainly due to the fast adsorption and desorption rate of water particles on the surface of the CMFTO electro-ceramic.

The CMFTO electro-ceramic based humidity sensor showed a maximum hysteresis of about 3.2% corresponding to 85% RH (see Figure 10). This result indicates that the hysteresis of our developed CMFTO electro-ceramic based humidity sensor is relatively lower than that of other different capacitive humidity sensors (i.e., ~4.16% for ZnO/Si-based, ~4.5% for SiC nanowire-based, ~4% for alumina nanowire-based, ~5% for graphene oxide-based, ~12% for (PEPC + NiPc + Cu_2_O)-based and so on) developed by other studies reported elsewhere [16,54,59,60,66]. The lower hysteresis value was obtained owing to the relatively faster adsorption and desorption rate of water molecules on the surface of the CMFTO electro-ceramic in comparison to the other materials [54,55].

**Figure 11:**
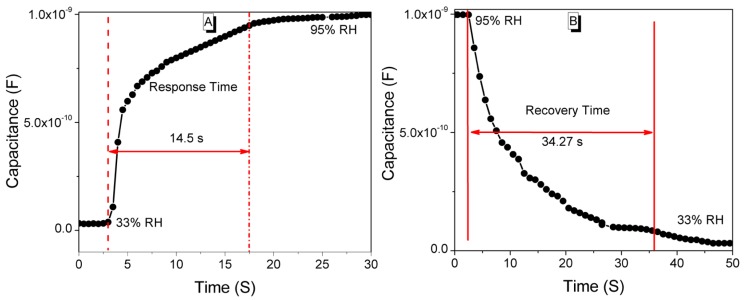
Response and recovery times of the CMFTO humidity sensors for humidity levels between 33% RH and 95% RH at 10^2^ Hz. (**A**) Response time (14.5 s); (**B**) Recovery time (34.27 s).

The response and recovery time have a significant effect on the performance of humidity sensors. Time taken by a sensor to achieve ~90% of the total capacitance change is defined as the response time in case of adsorption or the recovery time in case of desorption of the water vapors. For a good sensor, the response and recovery time of humidity sensors must be very small. The response (see Figure 11A) and recovery (see Figure 11B) characteristic curves of our CMFTO electro-ceramic based humidity sensor show a change in capacitance with time in seconds. From the plots, it has been observed that the response and the recovery times of the sensor were 14.5 s and 34.27 s, respectively. The obtained response and recovery times of the CMFTO electro-ceramic-based capacitive humidity sensor are better than those of other conventional capacitive sensors. For example, the response time of our present sensor based on CMFTO electro-ceramic is significantly better than some humidity sensors based on other materials such as silicon nanowires (32 s in 11.3%–93% RH), anodic aluminum oxide (188 s in 30%–95% RH), multi-wall carbon nanotubes (45 s in 11%–86% RH), macroporous silicon (20 min in 0%–100% RH), and so on [67,68,69,70]. Both the response and recovery times of the present material are also better than those of Pb-free BaTiO_3_ based nanomaterials (i.e., 15 and 120 s, respectively, in 33%–98% RH) [71].

**Figure 12:**
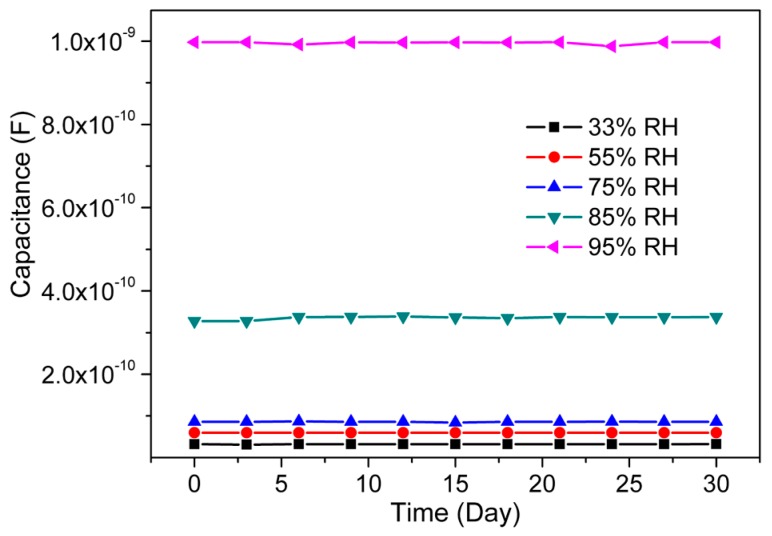
Stability analysis of CMFTO electro-ceramic-based humidity sensor measured at a test frequency 10^2^ Hz at 25 °C. Note: The measurement was conducted repeatedly for 30 days at 2-day interval and very negligible changes are observed.

Stability is one of the most important parameter for any sensor application. The present CMFTO electro-ceramic-based humidity sensor was tested repeatedly at 10^2^ Hz under fixed humidity levels (33%, 55%, 75%, 85% and 95% RH) for a period of 30 days and its humidity sensing parameters were measured repeatedly every 2 days. The characteristics of the present CMFTO sensor depicted in Figure 12 exhibit a good stability since there is only slight variation in capacitance as time goes by. Therefore, the obtained stability characteristics confirm that CMFTO sensor is a good promising material for practical applications.

**Figure 13:**
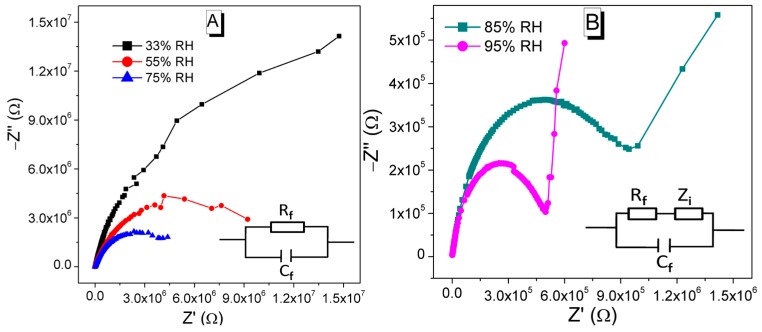
Complex impedance plots and equivalent circuits of CMFTO based electro-ceramic under different humidity levels. (**A**) At lower humidity range (33%–75% RH), single semicircles are formed; the inset represents an equivalent circuit at lower RH; (**B**) At higher humidity condition (85%–95% RH), the radii of semicircle decrease and a straight line appears, and the straight lines become longer with increasing of humidity; the inset represents an equivalent circuit at higher RH. *R*_f_ and *C*_f_: are the resistance and capacitance of CMFTO electro-ceramics, respectively; *Z*_i_: interface impedance between CMFTO electro-ceramic surface and electrode.

Complex impedance spectroscopy is a powerful tool to understand the polarization processes and that take place in a humidity sensor due to absorption of water particles and the conductivity mechanism. The Nyquist/complex impedance plot of the CMFTO-based humidity sensor at different RH (33%–95% RH) was measured over a frequency range of 10^2^ Hz to 10^6^ Hz with 1 V AC signal at 25 °C. The variation in impedance spectra suggests different water absorption mechanisms related to electrical conductivity and polarization occur in the CMFTO electro-ceramic. When the RH is low (33%–75% RH), a half semicircle is observed in the complex impedance plot as depicted in Figure 13A. These semicircle characteristics can be explained by the resistance-capacitance parallel electrical circuits as shown in Figure 13A (inset). Therefore, the intrinsic resistance of the sensing electro-ceramic is responsible for the formation of the semicircle. The curvature of the semicircle decreases with increasing RH and results the decrease in intrinsic impedance, which is mainly due to the interaction between the sensing nanomaterial and water molecules. The polarization processes and conductivity mechanism that take place in a humidity sensor can be expressed by the resistance and capacitance electrical model as well by ionic interface and diffusion phenomena. With increasing RH (85%–95% RH), a straight line is generated in the low-frequency range and the semicircle becomes small (Figure 13B). The ionic as well as electrolytic conductivity are mainly responsible for the formation of a straight line in the complex impedance plot [72,73]. The straight line connected semicircular response curve represents another type of sensing mechanism which can be modelled by a capacitive and resistive equivalent electrical model as depicted in Figure 13B (inset) [72,73]. In these equivalent circuits of such complex impedance plots, R_f_ stands for the resistance of the CMFTO electro-ceramic, which decreases as RH increases, *C*_f_ is the capacitance of the CMFTO electro-ceramic and *Z*_i_ is the interfacial impedance at the electrodes/CMFTO electro-ceramic [54,74]. At very low RH, the capacitive behaviour is mainly attributed to the conductivity of protons and the resistive characteristic is related to H_3_O^+^ ions. When the RH increases gradually, a huge number of water molecules participate in the physisorbed process on the large specific surface area of the CMFTO electro-ceramic. Thus, proton hopping between the adjacent water molecules occurs easily in CMFTO electro-ceramic. This causes a decrease in the intrinsic resistance of the CMFTO electro-ceramic corresponding to the more depressed semicircles and longer straight lines in Figure 13B at higher RH values. As higher the % RH, the line becomes longer and the semicircle becomes smaller. The line represents the Warburg impedance and occurs due to the diffusion of the electroactive species at the electrodes [74]. Hence, the complex impedance plots represent a single semicircle appears at lower humidity (33%–75% RH) (Figure 13A) and a straight line appears just after the 75% RH (Figure 13B). The single semicircle and the semicircle with straight line show completely two different sensing mechanisms. It was also clearly observed in Figure 9 where two different slopes of logC vs. RH plot contributed to the change in adsorption mode of water molecules from a monolayer chemisorption at low RH and a multilayer physisorption at high RH. It is thus indicated that the 75% RH is a critical point for water absorption mechanism of the present CMFTO and it is clearly confirmed that the two different conduction mechanisms take place at the surfaces of CMFTO electro-ceramic.

## 4. Conclusions

A novel submicroporous Ca,Mg,Fe,Ti-Oxides electro-ceramic-based capacitive humidity sensor has been fabricated from some inexpensive oxide nanomaterials using a solid-state step-sintering process. The presented sintering technique helped get nanomaterials with the desired morphology, lower density and high porosity. This newly developed Pb-free CMFTO electro-ceramic has shown improved capacitive humidity sensing properties via physisorption. It has shown two distinct conduction mechanisms by displaying a critical RH at 75% RH. The capacitance of the sensor of the CMFTO electro-ceramic increased from 3.2183 × 10^−11^ F to 9.9741 × 10^−10^ F as the RH changed from 33% to 95% at a testing frequency of 10^2^ Hz. It has shown a high sensitivity (3000%), rapid response (14.5 s) and recovery (34.27), which is much lower than the values of other conventional capacitive humidity sensors. The present capacitive sensor showed a very low hysteresis of 3.2%. Therefore, all the above improved sensing characteristics, together with the good linearity and stability of the CMFTO electro-ceramic indicate that it can be used as a potential humidity sensing material for advanced applications.

## Figures and Tables

**Table 1:** Schematic sintering steps of the samples 450, 650, 850, and 1050 °C.

Steps	Parameters	Sample Details			
		450 °C	650 °C	850 °C	1050 °C
Step-I	Temperature (°C)	450	250	350	350
	Time (h)	3.5	1	1	1
	Ramp rate (°C/min)	5	5	5	5
Step-II	Temperature (°C)	-	650	550	550
	Time (h)	-	3.5	3.5	3.5
	Ramp rate (°C/min)	-	10	10	10
Step-III	Temperature (°C)	-	-	850	1050
	Time (h)	-	-	1.3	1.3
	Ramp rate (°C/min)	-	-	15	15
Step-IV	Temperature (°C)	-	-	750	750
	Time (h)	-	-	3	3
	Ramp rate (°C/min)	-	-	20	20

## Supplementary Materials

Supplementary File 1
