# Supplementary material for: Design and Development for Capacitive Humidity Sensor Applications of Lead-Free Ca,Mg,Fe,Ti-Oxides-Based Electro-Ceramics with Improved Sensing Properties via Physisorption
Source: Sensors (Basel). 2016 Jul 21;16(7):1135. doi: 10.3390/s16071135 (PMC4970177; doi:10.3390/s16071135)
Supplement: Supplementary File 1 [file sensors-16-01135-s001.pdf]

Supplementary Material: Design and Development for Capacitive Humidity Sensor Applications of Lead-Free Ca,Mg,Fe,Ti-Oxides-Based Electro-Ceramics with Improved Sensing Properties via Physisorption

Ashis Tripathy, Sumit Pramanik, Ayan Manna, Satyanarayan Bhuyan, Nabila Farhana Azrin Shah, Zamri Radzi and Noor Azuan Abu Osman

Morphological and Structural Analysis

Morphological Study

Average particle size of the unsitered ceramic (~200 nm, see Figure S1a) was found to increase after sintering at 450 °C (~350 nm, see Figure S1b) and 650 °C (~750 nm, see Figure S1c). However, particle size growth rate was controlled despite further increases of temperature from 850 and 1050 °C without much change in total pore size distribution, where average pore sizes are 500 and 850 nm, respectively. The average particle sizes of CMFTO samples sintered at 850 and 1050 °C are 780 and 670 nm, respectively (see Figure S1d,e). A smaller size (typically < 100 nm) of new phase of $CaTiO_3$ particles have been noticed in Figure S1e.

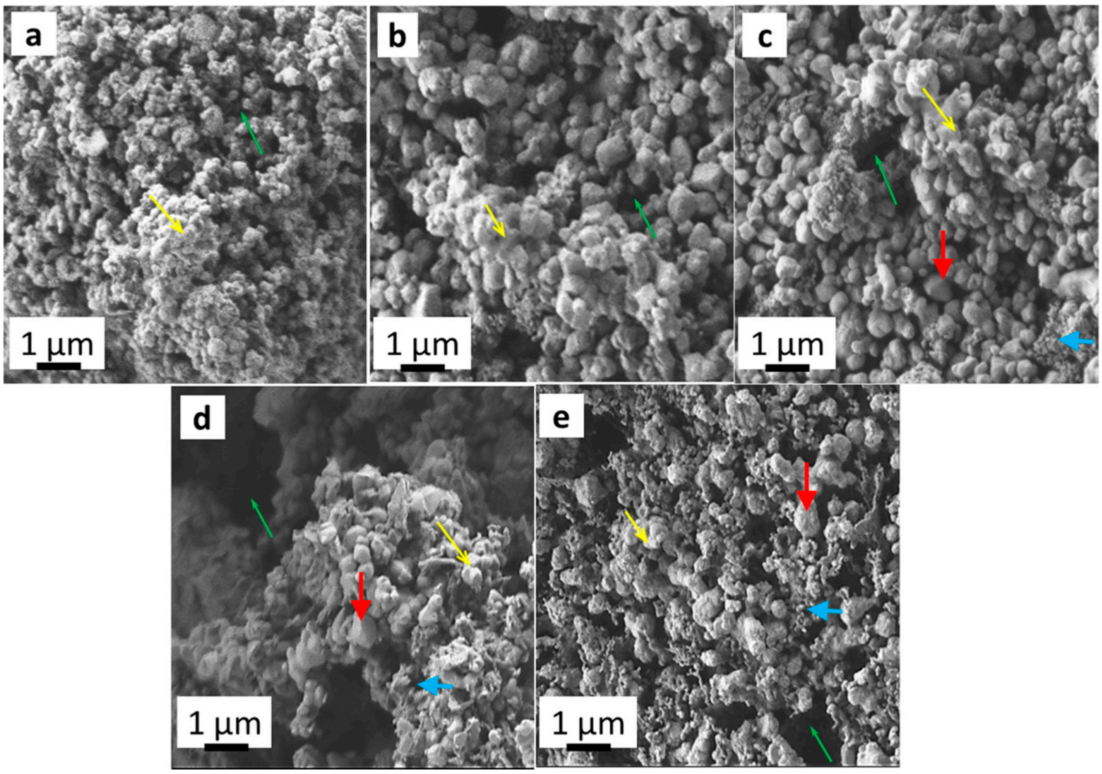

Figure S1. Scanning electron micrographs of the specimens for (**a**) unsintered; and sintered at (**b**) 450 °C; (**c**) sintered at 650 °C; (**d**) sintered at 850 °C; and (**e**) sintered at 1050 °C. Note: Green arrows indicate pores, yellow arrows indicate particles, vertical red arrows indicate armalcolite phase and horizontal blue arrows indicate perovskite phase.

X-ray Diffraction (XRD)

The crystalline X-ray diffraction (XRD) peaks of all the used raw materials are depicted in Figure S2a for unsintered ceramic. XRD patterns of the sintered samples are directed in Figure S2b–e. After sintering at 450 °C, it was started to convert into a new phase of armalcolite ($Fe_2MgTi_3O_{10}$). With further rising of temperatures crystalline peaks of another phase of perovskite $CaTiO_3$ have been revealed. Then, the XRD peaks of $Fe_2MgTi_3O_{10}$ and $CaTiO_3$ phases become more prominent after sintering at 1050 °C as they are clearly seen in Figure S2. A small crystalline peak of Fe_3O_4 due to (110) plane at $2\theta = 35.70°$ has been observed in the material after sintering at 1050 °C (see Figure S2e).

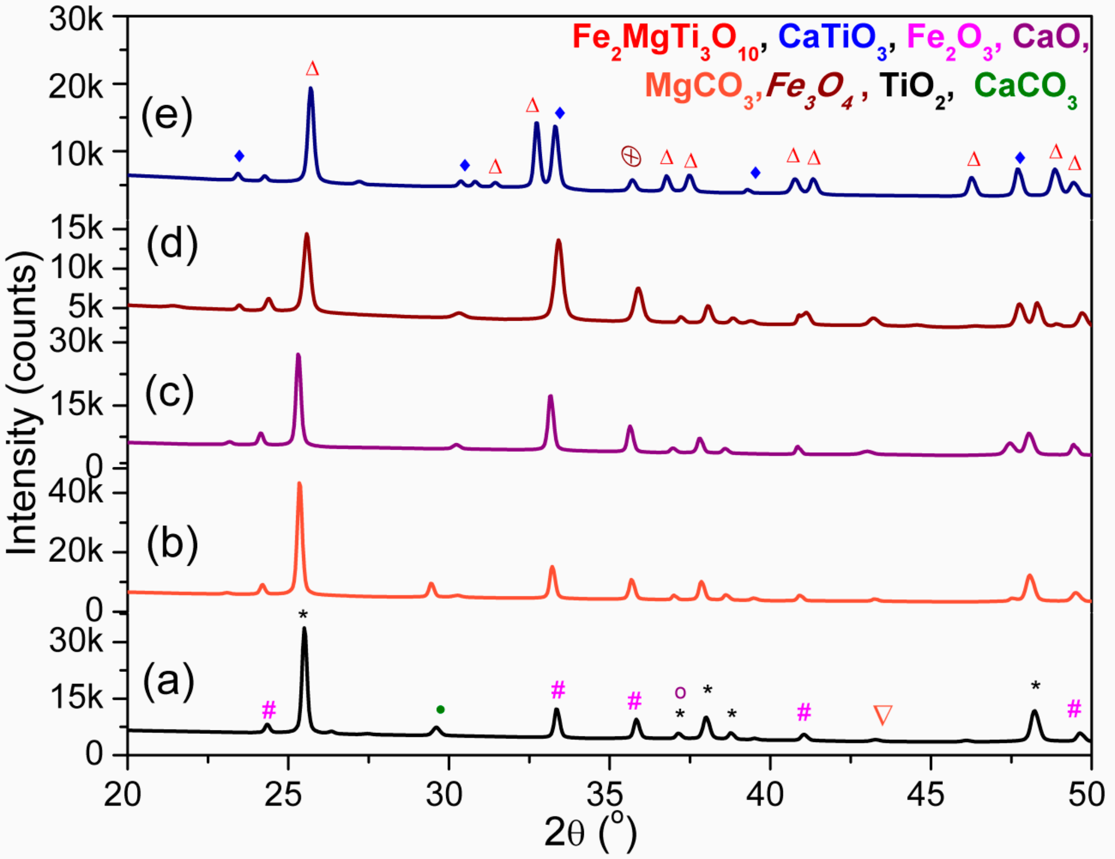

Figure S2. X-ray diffraction study of (**a**) unsintered and sintered at (**b**) 450 °C, (**c**) 650 °C, (**d**) 850 °C, and (**e**) 1050 °C samples. Note the different coloured planes denote the crystalline planes of corresponding materials: pink-hash—Fe_2O_3, black-star—TiO_2, orange-opened inverted triangle—$MgCO_3$, violet-opened circle—CaO, green-closed circle—$CaCO_3$, red-opened triangle—$Fe_2MgTi_3O_{10}$, brown-plush enclosed by oval—Fe_3O_4, and blue-closed diamond—$CaTiO_3$.
